# Origins and History of the Minimal Model of Glucose Regulation

**DOI:** 10.3389/fendo.2020.583016

**Published:** 2021-02-15

**Authors:** Richard N. Bergman

**Affiliations:** Diabetes and Obesity Research Institute, Cedars-Sinai Medical Center, Los Angeles, CA, United States

**Keywords:** diabetes, metabolism, mathematical model, disposition index, insulin clearance, glucose effectiveness

## Abstract

It has long been hoped that our understanding of the pathogenesis of diabetes would be helped by the use of mathematical modeling. In 1979 Richard Bergman and Claudio Cobelli worked together to find a “minimal model” based upon experimental data from Bergman’s laboratory. Model was chosen as the simplest representation based upon physiology known at the time. The model itself is two quasi-linear differential equations; one representing insulin kinetics in plasma, and a second representing the effects of insulin and glucose itself on restoration of the glucose after perturbation by intravenous injection. Model would only be sufficient if it included a delay in insulin action; that is, insulin had to enter a remote compartment, which was interstitial fluid (ISF). Insulin suppressed endogenous glucose output (by liver) slowly. Delay proved to be due to initial suppression of lipolysis; resultant lowering of free fatty acids reduced liver glucose output. Modeling also demanded that normalization of glucose after injection included an effect of glucose itself on glucose disposal and endogenous glucose production – these effects were termed “glucose effectiveness.” Insulin sensitivity was calculated from fitting the model to intravenous glucose tolerance test data; the resulting insulin sensitivity index, SI, was validated with the glucose clamp method in human subjects. Model allowed us to examine the relationship between insulin sensitivity and insulin secretion. Relationship was described by a rectangular hyperbola, such that Insulin Secretion x Insulin Sensitivity = Disposition Index (DI). Latter term represents ability of the pancreatic beta-cells to compensate for insulin resistance due to factors such as obesity, pregnancy, or puberty. DI has a genetic basis, and predicts the onset of Type 2 diabetes. An additional factor was clearance of insulin by the liver. Clearance varies significantly among animal or human populations; using the model, clearance was shown to be lower in African Americans than Whites (adults and children), and may be a factor accounting for greater diabetes prevalence in African Americans. The research outlined in the manuscript emphasizes the powerful approach by which hypothesis testing, experimental studies, and mathematical modeling can work together to explain the pathogenesis of metabolic disease.

## Early Thoughts and Personal Issues

**Figure 1:**
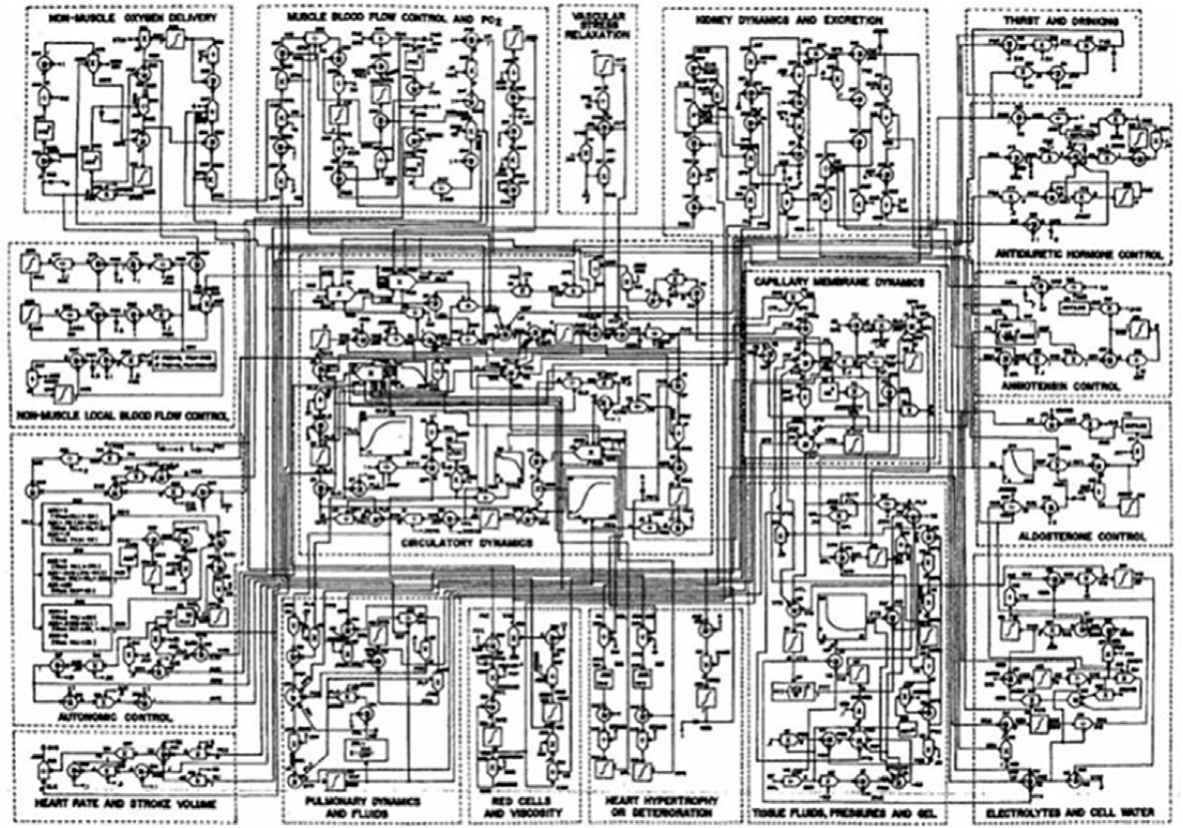
Guyton model of the cardiovascular system.

Mathematical modeling of physiological systems gained interest in the early 1950’s. One of the earliest models in the metabolic field was that of Bolie, who represented the glucose/insulin relationship in terms of two simple equations (1). During the same period, more complex models were introduced. One example is Guyton’s model of the cardiovascular system (2). It was Guyton’s goal to include all (at the time) known information regarding the known physiology of the cardiovascular system, and he included additional interactions which emanated from his own work (**Figure 1**). While Guyton and colleagues were able to gain much insight from this work, the model was not usable by the scientific or medical communities, in view of its great complexity.

My own background was as an electrical engineer. I was virtually without training in the biological sciences. This changed due to interaction with Professor Oscar Hechter (my uncle by marriage) of the Worcester Foundation of Experimental Biology. Hechter suggested I contact John Urquhart of the University of Pittsburgh; John along with F. Eugene Yates, were pioneers of modeling of endocrine systems. Their electromechanical model of the adrenocortical system remains a classic (3). I joined Urquhart’s lab despite my lack of education in the biological sciences. He was patient, and he taught me much about experimental physiology. At Pitt, I came in contact with I. Arthur Mirsky, who was a giant of the field of carbohydrate metabolism. I made a major life decision; I believed that mathematical modeling of carbohydrate metabolism could in the end be even more important for patient care.

**Figure 2:**
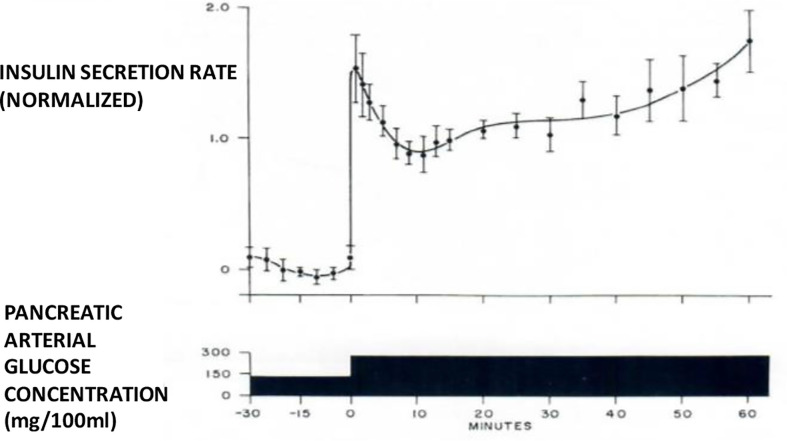
Biphasic insulin release from perfused pancreas.

**Figure 3:**
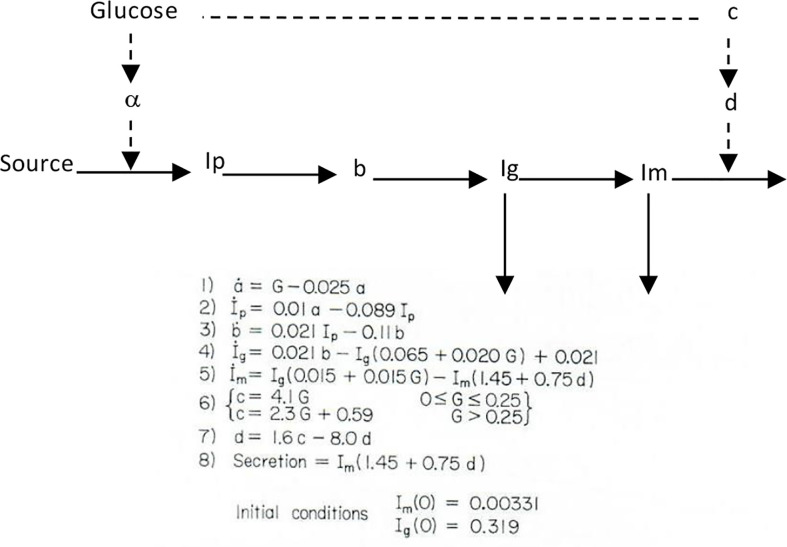
Bergman model of insulin secretion.

I therefore chose to study, for my PhD thesis, not modeling of the adrenocortical system, but modeling of the endocrine pancreas. I developed the cross-perfused pancreas system so I could measure the *dynamics* of insulin release from the endocrine pancreas (4). In fact, I believe that we were the first to discover that insulin release from the isolated pancreas was *biphasic* (**Figure 2**). Gerald Grodsky confirmed this result in the rat (5). For my PhD thesis, I developed one of the first mathematical models of pancreatic insulin secretion (**Figure 3**).

I was unfortunate (probably fortunate) that my PhD advisor abandoned our lab, setting me out for the first time as a truly independent investigator, although I was still a 22-year-old graduate student. I later followed John Urquhart to the University of Southern California Department of Biomedical Engineering. (It was very difficult to publish modeling papers in the standard endocrine or physiology literature at the time).

## Origin of the Minimal Model

In the context of “Frontiers in Physiology,” it is of interest to remember the resistance of the endocrine community to models in general. In fact, Departments of Physiology (at least in the United States) were highly suspicious of modeling studies in the 1970s. In part, this was due to a history of investigators who would propose models, but not test them in the laboratory (6); such models often “died on the vine”. Personally, I was determined to combine modeling with rigorous experimental testing—an approach our laboratory continues to apply to this day. (I identified with George Gershwin, dedicated to make a “Gentleman out of Jazz”. Maybe we could help make a “Gentleman out of Modeling” in carbohydrate regulation).

During the first decade of my independent laboratory (1971–1980), we introduced several disparate models, such as a differential equation model of insulin secretion (discussed above), a “random hit” statistical representation of hormone binding and activation (7), and a differential equation model of liver glycogen metabolism (8). However, one model that has survived the test of time is the so-called “minimal model” of carbohydrate metabolism.

**Figure 4:**
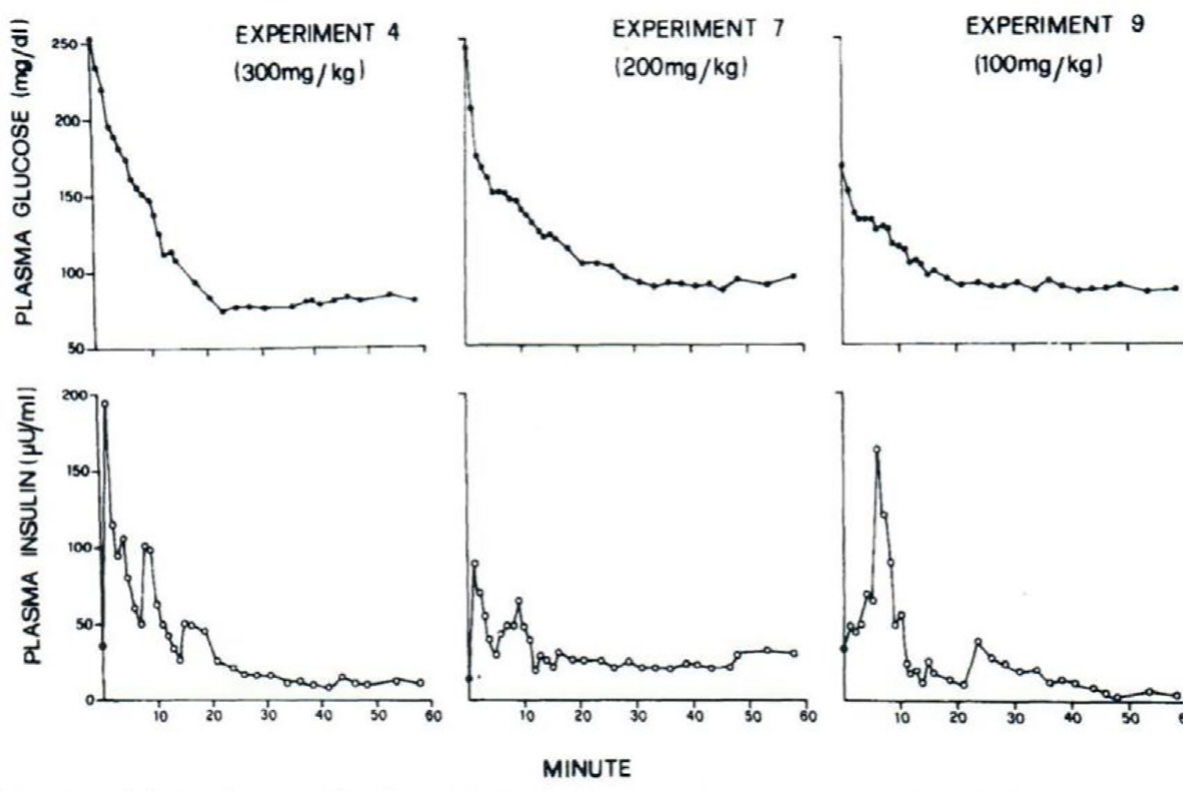
Early IVGTTs with frequent blood sampling.

**Figure 5:**
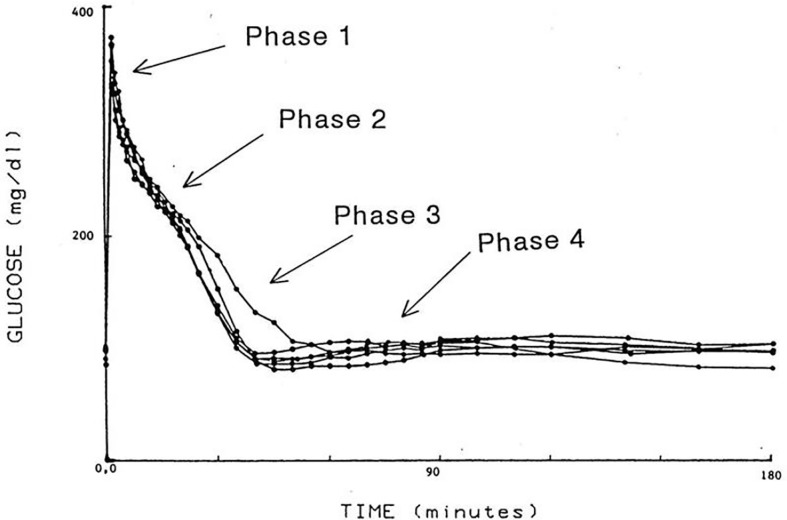
IVGTT phases.

I was approached by Alberto Salvan at the International Endocrine Society meeting in Copenhagen and invited to visit Padova, Italy. Alberto was sent by Claudio Cobelli, the young “star” of the Bioengineering Department at the University of Padova. I went to Padova and introduced Claudio to my original concept—I argued that previous models of physiological systems (particularly carbohydrate metabolism) were less than useful because they were either too complex (their parameters could not be uniquely specified from data) or too simplistic to accurately account for the data available. I also argued that the effort had not yet been put forth to obtain data which made it possible to make intelligent model design. Thus, in my laboratory at Northwestern University (I was there in Bioengineering from 1976 to 1979), I encouraged my graduate student, Y. Ziya Ider, to obtain a data set which we could use as a *basis* for modeling the regulation of the glucose level. At the time, the clinical tests of metabolism included the oral glucose tolerance test and the intravenous glucose tolerance test (IVGTT); both included glucose ingestion—oral or intravenous infusion—with infrequent (~1/h) sampling. We reasoned that more frequent sampling was necessary to reveal the actual patterns of glucose and insulin which resulted from carbohydrate administration. Indeed, performing the IVGTT and *sampling every minute for 180 min* (**Figure 4**) revealed that the time course of glucose and insulin after intravenous administration was more complex than revealed by the previously used hourly sampling (9). This choice of frequent sampling after glucose injection was a critical choice. It revealed that the return of glucose to basal (by 180 min) could be described by four temporal phases (**Figure 5**): a mixing phase of glucose in plasma, a quasi-exponential phase (see below, “glucose effectiveness”) an *acceleration* of the glucose decline (reflecting the action of insulin) followed by glucose’s return to pre-injection value (10).

## Concepts Underlying the Minimal Model

With this limited data base (**Figure 4**), Claudio Cobelli came to Evanston IL, and we began to build the model. This was a critical period; the manuscript emerging from the 6 weeks of work in the summer of 1978 was entitled “Quantitative estimation of insulin sensitivity (9)“. It is of interest that this seminal paper has been cited over 2,000 times; ironically, it remained virtually uncited for the first 10 years after publication.

Our basic goal was to find a “minimal model”. This would be a mathematical construct which was 1) based upon known physiological principles, 2) sufficiently complex to account for the intravenous data we obtained in our laboratory, and 3) simple enough that the model parameters could be calculated *from a single IVGTT performed in a single individual.*

## Partition Analysis

**Figure 6:**
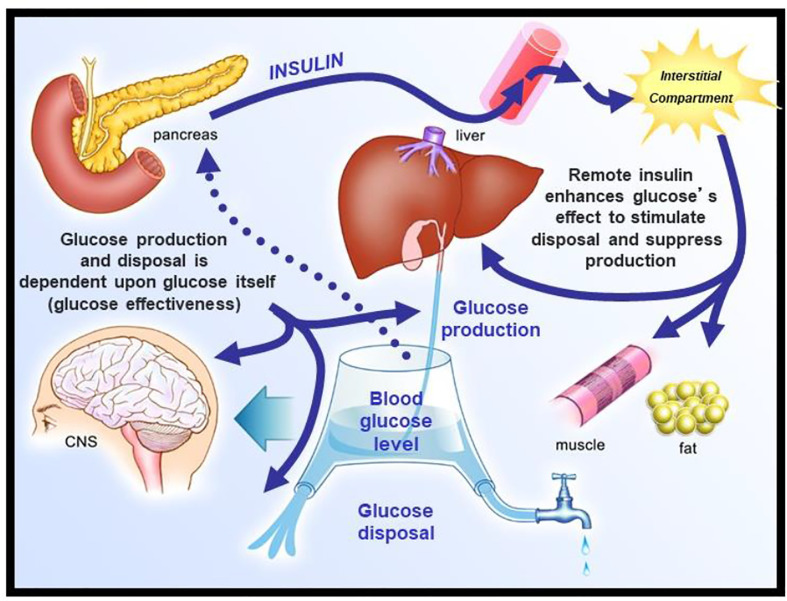
Closed loop system of glucose regulation.

We envisioned glucose regulation as a closed loop system (**Figure 6**), including glucose production and uptake, and insulin release from the pancreatic β-cells. Glucagon was not included in our original representation. However, we faced a serious dilemma: we knew from our previous work that it would be a great challenge to model insulin release from the endocrine pancreas. Therefore, we applied the principle of “partition analysis (11)“; we would treat the plasma insulin concentration as an “input” to the tissues producing and utilizing glucose, and the plasma glucose as the “output,” reflecting the effect of the known insulin on the turnover of glucose. This approach allowed us to model just the insulin-sensitive tissues, while obviating the difficult problem of modeling insulin secretion from the β-cells.

## Choice of the Model

**Figure 7:**
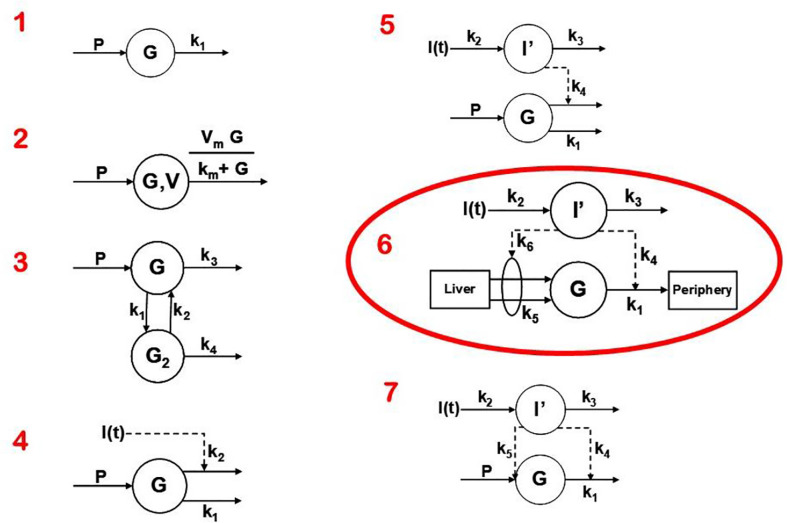
Models tested to determine “optimal” degree of complexity. Model 6 was chosen and was designated the “minimal model.”

Two approaches were possible—defining a complex model (representing all known physiology) and simplifying it, or choosing the simplest conceptual model, asking if it could account for the known data (**Figure 4**), and systematically introducing complexity until a best model could be found. The models we tested are in **Figure 7**. Note that the simplest model was glucose first order decay with no explicit insulin action; in Model 2 Michaelis Menten disappearance was added. Two compartment glucose distribution was added in Model 3. When we attempted to account for insulin glucose dynamics, we learned something very important: *it was not possible to account for glucose kinetics without a delay in insulin’s effects to increase glucose utilization and suppress glucose production*. As we shall see, further experimental studies which resulted from this realization that insulin’s effects were delayed in time had very important ramifications regarding insulin action *in vivo.* The model we finally chose, Number 6 in **Figure 7**, was therefore accepted as the minimal model of glucose utilization, and it remains the accepted model to this day.

**Figure 8:**
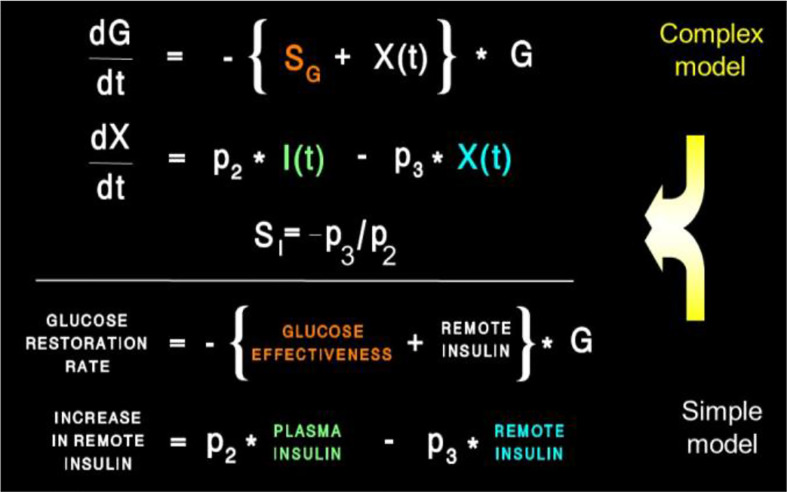
Equations of the minimal model and their “translation.”

**Figure 9:**
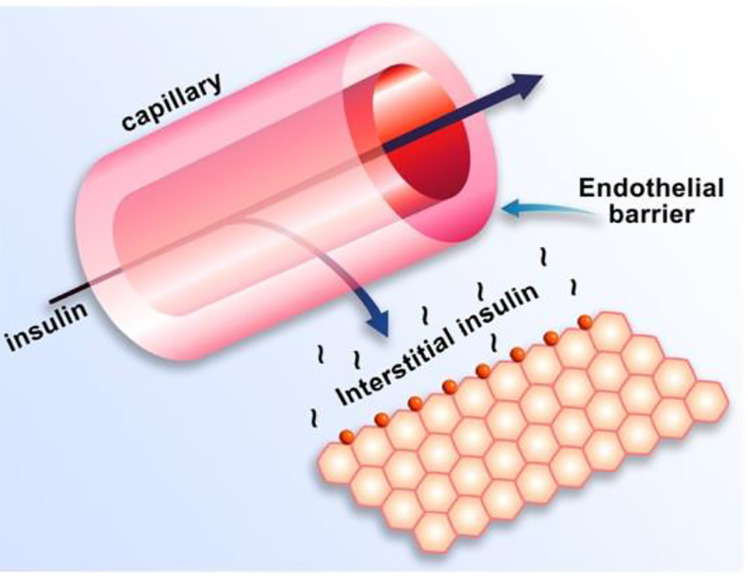
Schematic of insulin transport from blood to interstitial fluid.

Equations of the minimal model can be explained as follows (**Figure 8**): the model accounts for the return of glucose to the basal value after injection. As we had discovered that insulin’s effect had to be delayed, we assumed that insulin acts from a compartment *remote from plasma*. We hypothesized that the delay in insulin action could be due to a slow rate of movement of insulin from plasma to interstitial fluid (ISF), the latter bathing skeletal muscle. To test this concept, we performed a series of euglycemic clamp experiments in which we measured insulin in blood and in skeletal muscle lymph fluid, the latter as a surrogate of ISF (12–14). We discovered that the rate of glucose disposal was directly related to ISF insulin level, proving that the delay in insulin action *in vivo* is indeed explained by the slow transport of the hormone from the blood to the ISF (**Figure 9**).

One question that arose was why the modeling was acceptable with delays not only in insulin stimulation of glucose disposal (mediated by interstitial insulin) but also with *slow insulin suppression of endogenous glucose production (EGP).* It was known at the time that the binding of insulin to liver was very rapid. Why, then, was the effect of insulin to suppress glucose production similarly retarded as the disposal effect (15)? Possibly, insulin acted to suppress the liver not directly, but *indirectly via* a tissue remote from the liver. In fact, we hypothesized that insulin’s effect on the liver was mediated by free fatty acids (FFA); once insulin is infused, the hormone suppressed lipolysis in adipose tissue, and the resulting lowering of FFA acted to lower liver glucose production. In a series of studies carried out by Kerstin Rebrin and Garry Steil, we showed that not only was there a strong correlation between FFA suppression and the suppression of EGP, but that preventing the FFA suppression by infusion of intralipid prevented the decline in EGP (16, 17). Thus, we believe that the slow movement of insulin into ISF in adipose tissue was rate-limiting for the effect of insulin to suppress EGP; therefore, it was not necessary to include rapid suppression of EGP in the model to account for glucose dynamics *in vivo*.

Insulin kinetics in the minimal model are represented by equation 2; this first-order equation assumes that secreted insulin enters the ISF compartment where it is represented by variable “X,” which we now know to represent interstitial insulin. ISF insulin then exits the remote compartment by a first-order process. Glucose dynamics are represented in the first equation; the rate of return of glucose to basal following injection was envisioned to have an insulin-dependent component [in proportion to variable X(t), or ISF insulin]. Also, to model the data, it was *requisite* that glucose could return to basal also in proportion to its own concentration, driven by a term we referred to as parameter S_G_, which we named “glucose effectiveness.” *Glucose effectiveness is the ability of glucose* per se *to normalize its own concentration*. We showed that the minimal model was able to account for the dynamics of glucose observed after injection.

## Insulin Resistance

There has been a debate, going back decades, regarding the relative importance of insulin resistance versus β-cell failure in the pathogenesis of Type 2 diabetes mellitus. To address this issue, we felt it necessary to attempt to measure these factors from the glucose tolerance test. Applying the minimal model to the IVGTT, is it possible to access a measure of insulin resistance? Examination of the model (**Figure 8**) showed that two factors determined the ability of glucose to normalize after glucose injection—insulin action, represented by the parameter S_I,_ and glucose effectiveness (S_G_), which accounts for glucose’s ability to self-normalize. Represented mathematically, insulin sensitivity is given as the partial derivative of glucose disappearance on glucose and insulin. It was easy to demonstrate that this relationship results in the ratio of two parameters of the minimal model: p3/p2. Thus, we showed S_I_, the “insulin sensitivity index”, to be equal to the ratio of these parameters from the minimal model. This index appears in over 2,000 publications.

## Insulin Sensitivity Index: Is It Accurate?

**Figure 10:**
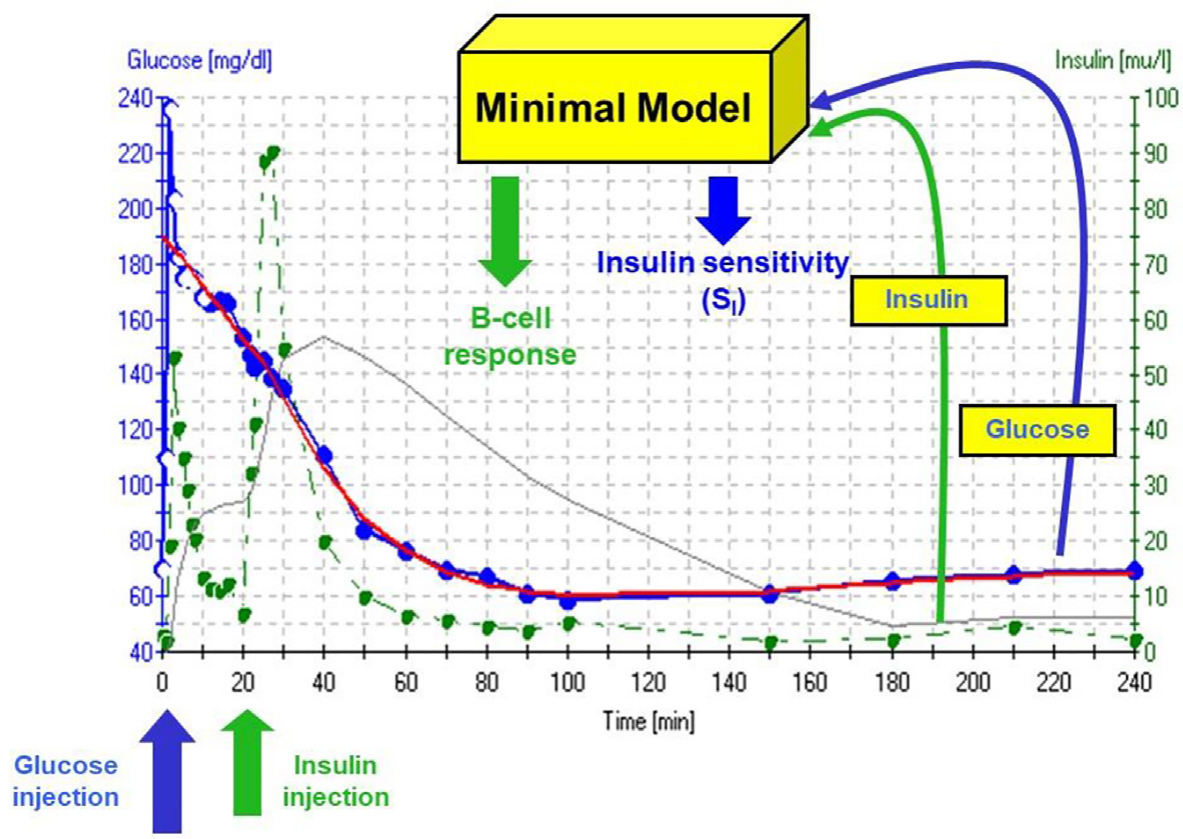
IVGTT protocol and minimal model output. Insulin data are”input” to the minimal model, which determines the best fit of the glucose dynamics and model parameters for that IVGTT.

The accuracy of the S_I_ was questioned by Reaven and colleagues (18). They claimed that in insulin resistant subjects, particularly insulin resistant patients with inadequate insulin response, the insulin sensitivity index from the minimal model correlated poorly with insulin sensitivity calculated from the euglycemic glucose clamp. Reaven’s manuscript, which appeared to be a blow to the minimal model method, was actually a godsend. We realized that a greater insulin pattern in blood would be necessary to accurately calculate insulin sensitivity from the IVGTT in resistant subjects. We therefore modified the IVGTT profile by adding an injection of the insulin secretagogue tolbutamide 20 min after glucose (**Figure 10**). Later the protocol was changed to inject insulin itself at 20 min after glucose, rather than tolbutamide (19).

## Validation of S_I_

It is generally assumed that the euglycemic glucose clamp (EGC) is the “gold standard” for the estimation of insulin sensitivity. Because most endocrinologists are not familiar with mathematical modeling, and may not trust modeling, it was of interest to validate the insulin sensitivity of the minimal model experimentally versus the clamp.

**Figure 11:**
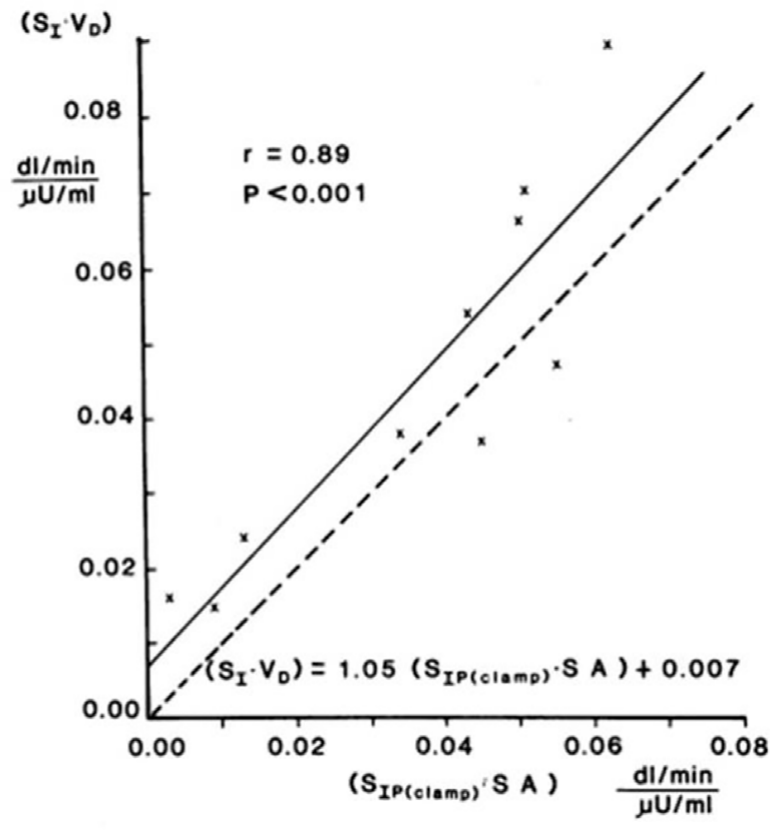
S_I_ equivalence between minimal model and clamp.

Validation studies were first carried out in the dog, where a significant correlation was observed between S_I_ and insulin sensitivity calculated from the EGC [r = 0.82, (20)]. This result was confirmed in human volunteers by Beard et al. (21). These studies alone supported the use of the IVGTT-based S_I_ for a *relative* measure of insulin sensitivity. However, the question naturally arose as to whether the IVGTT was measuring the *same physiological process* as the glucose clamp. Therefore, in collaboration with Jerrold Olefsky and colleagues, we compared minimal model values against the clamp (22). More important, we asked whether we could determine the IVGTT sensitivity values for a cohort of human subjects, and then determine what the clamp-based measures were in the same subjects. The strong correlation between IVGTT and clamp not only validated the IVGTT method, *but also demonstrated equivalency with the clamp*, when data from the two methods were expressed in identical units. We showed that insulin sensitivity from the clamp, defined as change in glucose disposal (ΔRd) induced with a measured change in plasma insulin (ΔI) per steady state glucose value [= ΔRd/(ΔI x G)], normalized by body surface area, was directly comparable to minimal model-derived S_I_ times the body distribution volume (S_I_ x V_D_). In fact, correlation in a group of individuals of varying body mass index was excellent; more important, the relationship had a slope not different from 1.0, and the regression line passed through the origin, demonstrating a lack of bias (**Figure 11**). These multiple validation studies supported the use of the IVGTT with minimal modeling as a potent tool to be used to study insulin action *in vivo* in large animals or human volunteers.

## The Disposition Index (DI)

**Figure 12:**
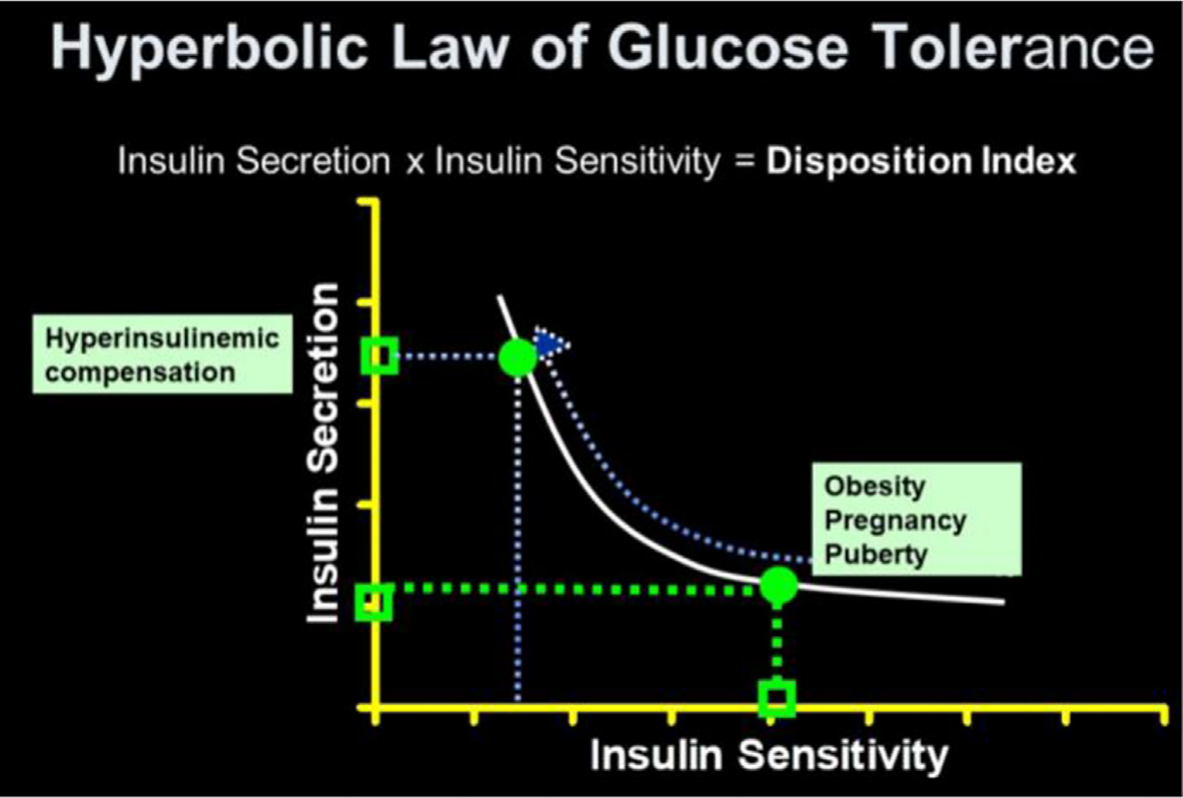
Disposition index (DI).

As previously stated, a debate regarding the relative importance of insulin resistance versus β-cell dysfunction in the pathogenesis of Type 2 diabetes raged for decades (23, 24). With the minimal model in hand, we hoped to contribute to help resolve this debate. We became interested not only in the measurement of insulin sensitivity and insulin release, *but the relationship between the two*. We hypothesized that in the face of insulin resistance, β-cell function would improve, and thus resist any change in glucose tolerance (**Figure 12**). We quantified this hypothesis as what became known as the “Hyperbolic Law of Glucose Tolerance (25)”.

This law can be stated as the following equation of a rectangular hyperbola:

$S_{I}\;\times \;AIR_{glucose}=\;DI$

in which S_I_ is insulin sensitivity, as discussed above, AIR_glucose_ is the first phase response of plasma insulin to the glucose injection, and DI was named the “Disposition Index”.

**Figure 13:**
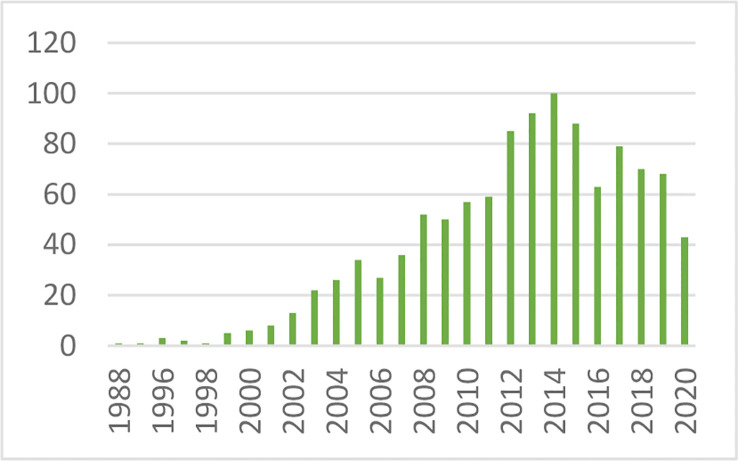
Cited publications pertaining to the DI.

After defining the hyperbolic relationship, we applied it to human subjects (26). It was shown that the *product* of insulin secretory response (which can be assessed as the first phase insulin response to glucose injection) multiplied by insulin sensitivity would be approximately constant in normal individuals. While initially controversial, the DI has now been accepted overwhelmingly by the diabetes community as the most accurate measure of β-cell function, and it has been cited in almost 1,000 publications as of this writing (**Figure 13**).

**Figure 14:**
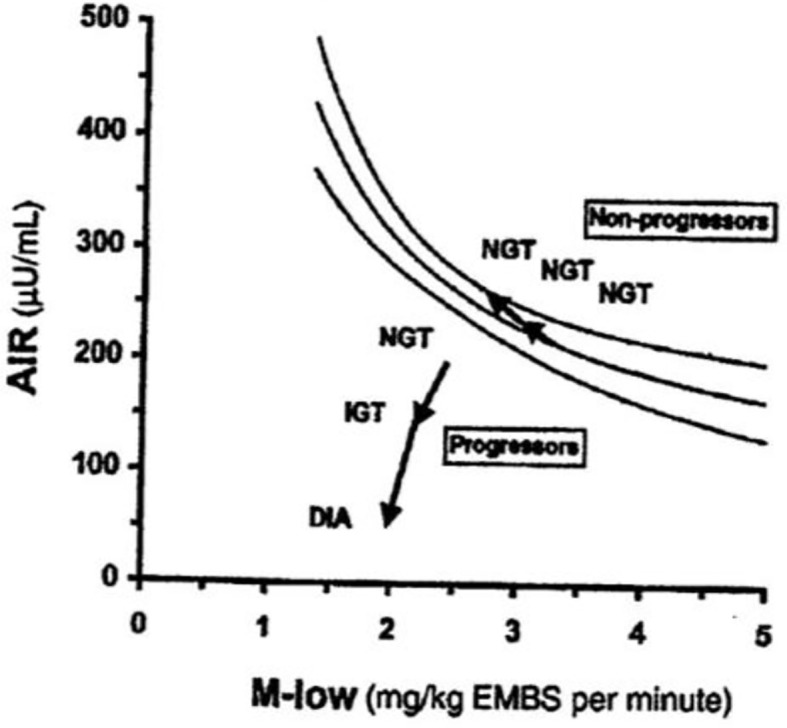
Predictive impact of DI on diabetes risk.

The DI represents the ability of the islet cells to *compensate* for insulin resistance. The resistance can be due to a variety of environmental changes, including obesity, pregnancy, and PCOS. The β-cells act to compensate, and under normal conditions prevent the frankly diabetic state. This is shown clearly in pregnancy, where severe insulin resistance in the third trimester is compensated by a massive islet cell response; glucose tolerance remains normal (27). Epidemiological studies have demonstrated that lower DI is a strong predictor of future diabetes (28, 29), and genetic studies have identified predictive variants related to DI (30). Weyer and colleagues showed in Pimas that lower DI predicts decline to Type 2 diabetes, whereas higher DI is protective [**Figure 14**; (31)]. It is of interest to remember that the DI emerged as a “child” of the minimal model itself; once it was possible to measure insulin sensitivity from the IVGTT, it was only natural to consider the relation to pancreatic islet cell function.

An unanswered question that remains is the underlying mechanism accounting for the hyperbolic relationship. We followed the development of enhanced insulin secretory response in normal dogs, demonstrating that the hyperbolic relationship is a *dynamic* one, as insulin response increased in proportion to insulin resistance (32). Experiments to identify the blood-borne signal responsible for the increase in insulin response suggested that nocturnal free fatty acids, peaking in the middle of the night, might provoke the enhanced secretory response, since blocking the nocturnal rise prevented the increment in the islet response (33); a similar mechanism is apparent in human volunteers (34). More data are needed to confirm or deny this latter mechanism in animals and in human subjects.

## Additional Factors

**Figure 15:**
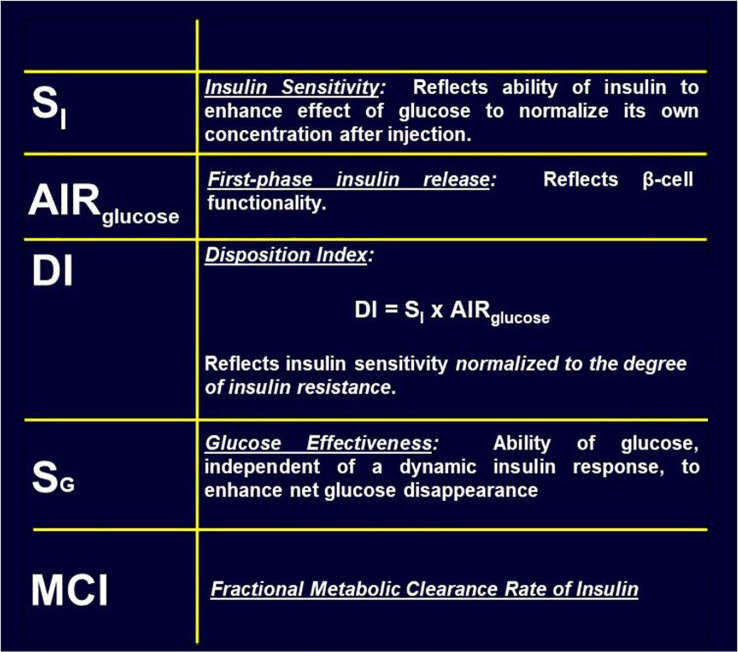
Factors contributing to glucose tolerance.

While historically focus was on insulin resistance and islet cell response, other factors can play a major role in the ability of the organism to utilize carbohydrate efficiently. Additional factors include insulin clearance and “glucose effectiveness” [**Figure 15**; (35, 36)]. Our laboratory has recently focused more on these additional factors. (Because our research has been based upon intravenous glucose administration, we have focused less on gastrointestinal agents such as GLP-1 and GIP).

## Insulin Clearance

Insulin is degraded primarily by liver and kidney. In fact, once secreted from the pancreas, about half the insulin presented to the liver *via* the portal vein is degraded and does not enter the systemic circulation. Our laboratory has recently considered the following question: “why would evolution choose to degrade half the secreted insulin before it has a chance to act to enhance glucose utilization by skeletal muscle and other tissues?” Working with the canine model allowed us to measure insulin clearance directly by comparing insulin infusion into the portal vein with systemic insulin infusion. Given matched infusion rates, the former route would result in less systemic insulin concentrations. By comparing insulin levels resulting from different routes of insulin administration, an accurate assessment of insulin clearance can be calculated (37, 38). We were surprised to discover a substantial variance in insulin degradation rates, even in normal animals; rates varied from 22 to 77% of portally presented insulin degraded during the initial pass through the liver (37).

**Figure 16:**
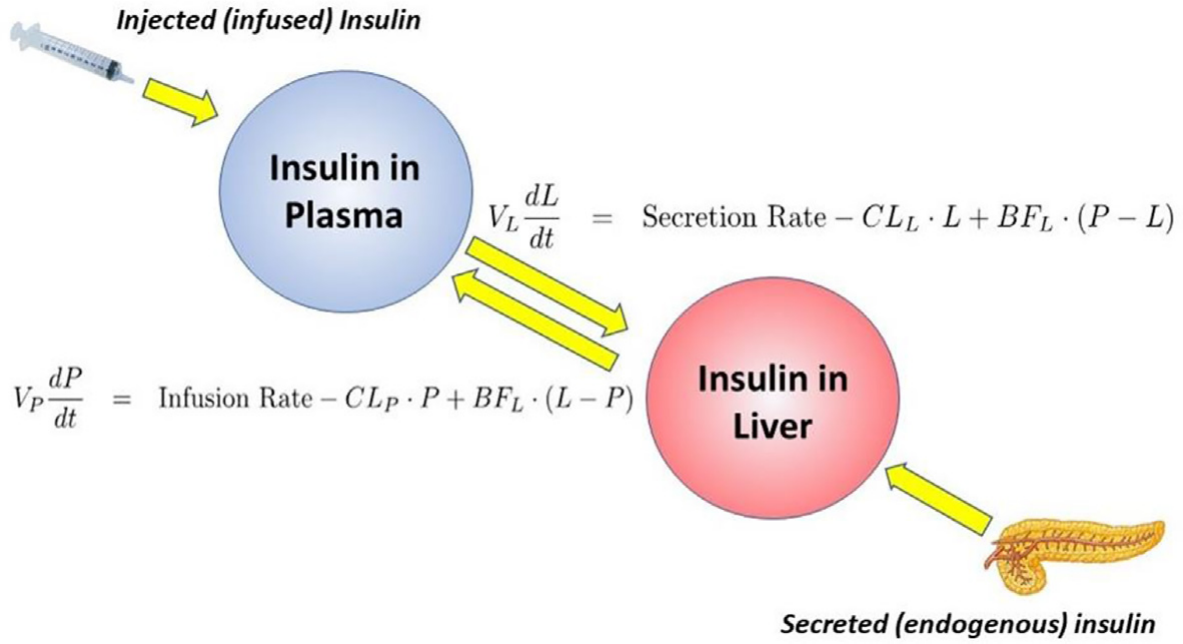
Model of insulin clearance.

**Figure 17:**
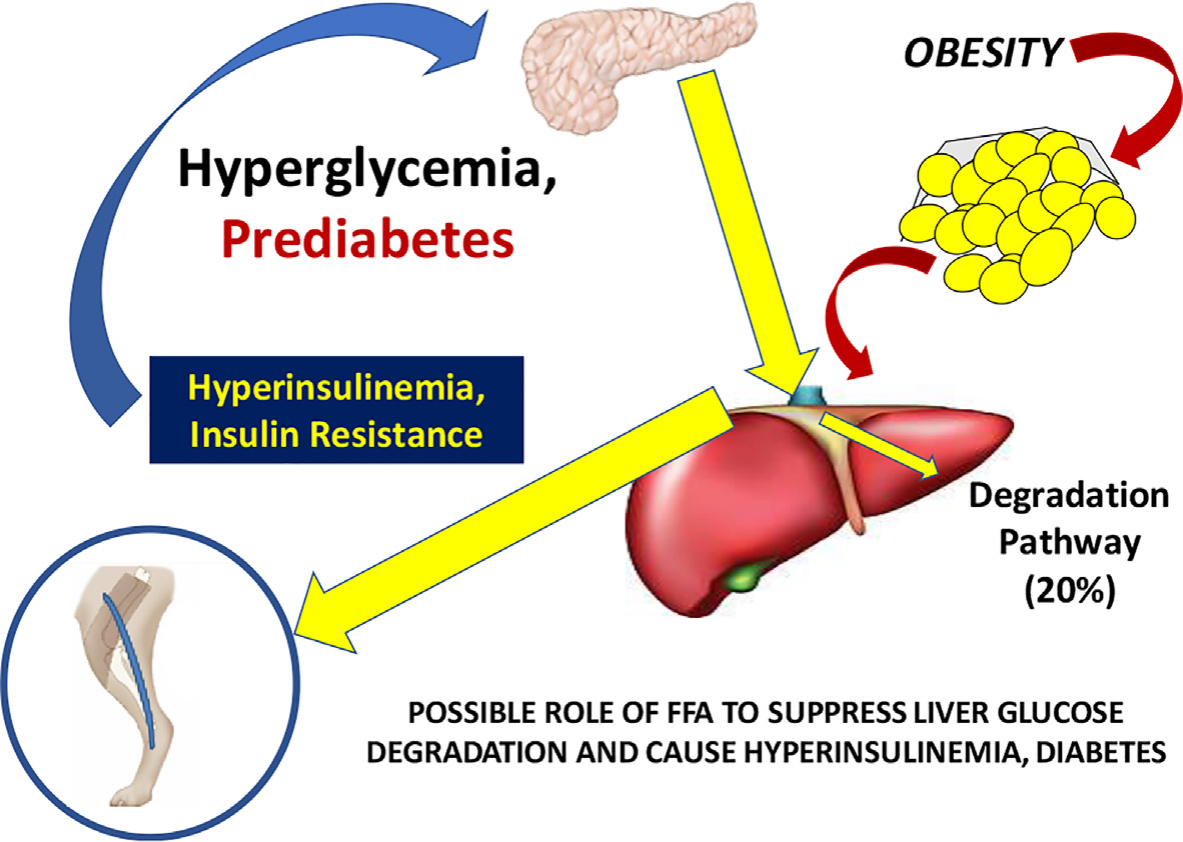
Hypothesis of the pathogenesis of Type 2 diabetes. It is suggested that increased plasma free fatty acids cause a reduction in hepatic insulin clearance, possibly by downregulation of IDE. A large proportion of insulin secreted by the β-cells therefore bypasses first-pass degradation, resulting in peripheral hyperinsulinemia. Higher plasma insulin downregulates skeletal muscle insulin sensitivity, stressing β-cells, and resulting in diabetes.

Working with David Polidori of Janssen Research, and Francesca Piccinini in our laboratory, we developed a new non-invasive model which allowed for estimation of first-pass hepatic clearance of insulin in human volunteers [**Figure 16**; (39)]. We were fortunate to obtain data from Drs. Barbara Gower and Jose Fernandez of the University of Alabama at Birmingham, which allowed us to apply our model to a human cohort from different ethnic groups (White, Hispanic American, African American), including nondiabetic adults and children, ages 7–13 years. In both adults and children, we confirmed that insulin clearance rates were significantly lower in African Americans than in Whites (40, 41). This lower insulin clearance can explain the hyperinsulinemia of African Americans (adults and children), which may contribute to the higher risk of Type 2 diabetes in those individuals. In our laboratory, we continue to examine the importance of variations in insulin clearance rates to diabetes risk, and mechanisms underlying the variations in clearance across different populations. While the mechanisms of insulin clearance, particularly in the liver, remain to be more clearly defined, it is apparent that insulin degrading enzyme (IDE) and CEACAM1 may both be involved (42). We have hypothesized that reduction in insulin clearance, particularly in liver, might be one cause of Type 2 diabetes, at least in some individuals. The concept is illustrated in **Figure 17**. Lower hepatic insulin clearance (in African Americans, for example) would result in a larger proportion of secreted insulin bypassing first-pass degradation of the hormone. This would result in systemic hyperinsulinemia, both at fasting and after nutrient intake. Hyperinsulinemia has been shown to downregulate insulin action in skeletal muscle (43, 44). The resulting insulin resistance would stress the pancreatic β-cells, potentially leading to prediabetes or diabetes itself (lower clearance, insulin resistance, and reduced β-cell function). While the putative importance of this hypothetical mechanism of diabetes pathogenesis remains to be proven, very recent data emerging from the NIH studies of diabetes in the Pima Nation appear to support this hypothesis. The NIH investigators, led by Douglas Chang, have very recently reported that in a study of 570 Pimas, followed over a period of 8 years, lower insulin clearance (measured by the glucose clamp) was a strong predictor of conversion from prediabetes to Type 2 diabetes mellitus, and this effect of lower insulin clearance was apparently independent of other factors (45). The NIH study appears to be a direct confirmation of the lower clearance hypothesis. However, further studies of the importance of insulin clearance in pathogenesis of diabetes remain to be done. Of particular interest is whether lower clearance is predictive in other ethnic groups, and what fraction of those who convert from prediabetes to diabetes may be due to reduced clearance, or other factors.

## Glucose Effectiveness

In the original choice of the minimal model (discussed above), we could only account for glucose normalization by including two fundamental processes: the effects of insulin to enhance glucose utilization (represented by factor S_I_) and a second term S_G_, which is the *effect of glucose per se to enhance glucose utilization independent of a dynamic insulin response.* We coined the term “glucose effectiveness” to describe this process, and while it is not totally understood, we continue to examine it. Marilyn Ader, in our laboratory, demonstrated the importance of S_G_ in experimental animals in studies where she demonstrated glucose’s ability to self-normalize (after injection) even if the dynamic insulin response is blocked (46).

**Figure 18:**
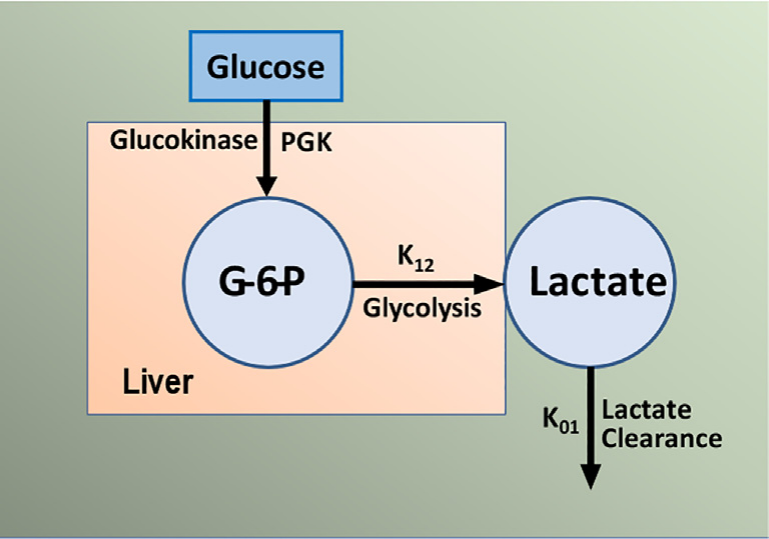
Simple model of glucose/lactate kinetics. Glucose enters hepatocytes, independent of insulin, and follows the glycolytic pathway via glucokinase. Lactate exits the liver and is a surrogate for glucokinase activation and “glucose effectiveness”.

The importance of S_G_ remains under investigation; we have proposed that it is a second defense for those at risk for Type 2 diabetes. Individuals with a combination of reduced insulin response and insulin resistance together can be protected from frank diabetes by a maintained glucose effectiveness. There has been some debate regarding the measurement of glucose effectiveness from analysis of the intravenous glucose tolerance test using the minimal model (47, 48). Inclusion of the secondary secretagogue, or insulin injection itself, during the test clearly improved the assessment of S_I_ but possible incorrect estimation of S_G_ is still a possibility. To improve this estimation, we have developed a new approach. The mechanisms underlying glucose effectiveness remain unclear, but we have proposed that much of the insulin-independent glucose utilization after carbohydrate intake is due to activation of hepatic glucokinase, resulting in a greater rate of glucose phosphorylation, glycogen deposition, and release of three-carbon intermediates from liver (predominately lactate). We have therefore developed a simple model of the liver, relating glucose uptake to lactate output from the liver (**Figure 18**). This model can be analyzed using data from the IVGTT, yielding an estimate of S_G_ independent of the traditional minimal model analysis of the IVGTT. We are presently evaluating the precision and accuracy of the “lactate model” approach (49).

## Commentary

It can no longer be doubted that mathematical modeling can have a great impact on our understanding of metabolic regulation. The minimal model is but one part of an extensive number of mathematical representations that have enabled the scientific community to understand metabolic physiology, to predict the time course of development of metabolic disease, and to design devices to more effectively regulate the blood sugar.

**Figure 19:**
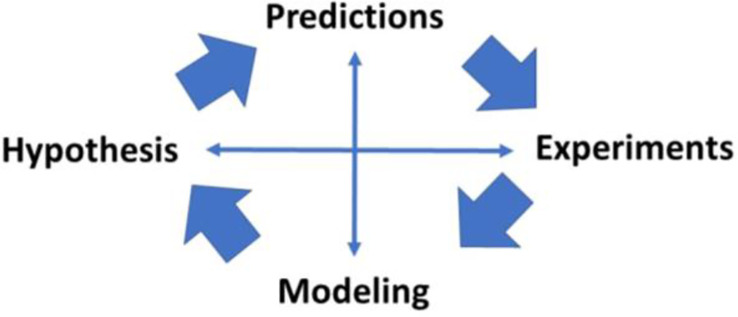
Importance of modeling in the scientific method.

The interaction among hypothesis, predictions, modeling and experimental testing of the models has characterized our work (**Figure 19**) and the work of other productive laboratories. It is of interest that investigations may begin at various points in the interactions shown in the figure; the minimal model itself began first with experimental data, then the model was proposed, and predictions of the model were tested in experimental models. In some cases, the model resulted in predictions (e.g., slow effect of insulin) which were examined in new experimental models (sampling of interstitial fluid). The possible role of insulin clearance in pathogenesis of Type 2 diabetes began with a hypothesis (lower clearance predicted diabetes) and examined with population studies (lower clearance in African American adults and children). Thus we have enjoyed, and we recommend, studying the interaction among these four activities to further our understanding of the mechanisms underlying metabolic disease at the organ level.

We were fortunate to have in our armamentarium the ability to model using modern computer techniques, and the availability of our laboratory to test our hypotheses directly. We were lucky to assemble a group of colleagues, drawn from biomedical engineering, mathematics, experimental physiology and molecular biology, to do our work. We can only thank them and the scientists with whom they worked for our modest success in helping to understand the complex but fascinating story of the regulation of carbohydrate metabolism in the intact organism.

## Data Availability Statement

The original contributions presented in the study are included in the article/supplementary material. Further inquiries can be directed to the corresponding author.

## Ethics Statement

The studies involving human participants were reviewed and approved by University of Alabama Human Subjects Committee. Written informed consent to participate in this study was provided by the participants’ legal guardian/next of kin. Animal studies were reviewed and approved by USC IACUC and Cedars IACUC.

## Author Contributions

The author confirms being the sole contributor of this work and has approved it for publication.

## Funding

This research was supported by research grants to Richard N. Bergman from the National Institutes of Health (DK 29867, DK 27619).

## Conflict of Interest

The author declares that the research was conducted in the absence of any commercial or financial relationships that could be construed as a potential conflict of interest.
